# Unlocking Platelet Mechanisms through Multi-omics Integration: A Brief Review

**DOI:** 10.2174/011573403X334382241210064101

**Published:** 2025-01-13

**Authors:** Muhammad Mazhar Fareed, Maryam Qasmi, Zarmina Khan, Haider Ali, Stavros Stavrakis, Carola Y. Förster, Sergey Shityakov

**Affiliations:** 1 Department of Computer Science, School of Science and Engineering, Università degli Studi di Verona, Verona, Italy;; 2 Applied Bioinformatics Group, Department of Biotechnology, Università degli Studi di Verona, Verona, Italy;; 3 Department of Computer Science, Systems, and Communications, Università degli Studi di Milano-Bicocca, Milan, Italy;; 4 Department of Bioinformatics and Biotechnology, Faculty of Life Sciences, Government College University, Faisalabad, Pakistan;; 5 Department of Meat Science and Technology, University of Veterinary and Animal Sciences, Lahore, Pakistan;; 6 Cardiovascular Section, Department of Medicine, University of Oklahoma Health Sciences Center, Oklahoma City, OK 73104, USA;; 7 Department of Anesthesiology, Intensive Care, Emergency and Pain Medicine, University Hospital Würzburg, Oberdürrbacher Str. 6, Würzburg, 97080, Germany;; 8 Laboratory of Chemoinformatics, Infochemistry Scientific Center, ITMO University, Saint Petersburg, Russian Federation

**Keywords:** Platelets, multiomics, biochemical methods, cardiovascular diseases, biomedicine, rational drug design

## Abstract

Platelets, tiny cell fragments measuring 2-4 μm in diameter without a nucleus, play a crucial role in blood clotting and maintaining vascular integrity. Abnormalities in platelets, whether genetic or acquired, are linked to bleeding disorders, increased risk of blood clots, and cardiovascular diseases. Advanced proteomic techniques offer profound insights into the roles of platelets in hemostasis and their involvement in processes such as inflammation, metastasis, and thrombosis. This knowledge is vital for drug development and identifying diagnostic markers for platelet activation. Platelet activation is an exceptionally rapid process characterized by various posttranslational modifications, including protein breakdown and phosphorylation. By utilizing multiomics technologies and biochemical methods, researchers can thoroughly investigate and define these posttranslational pathways. The absence of a nucleus in platelets significantly simplifies mass spectrometry-based proteomics and metabolomics, as there are fewer proteins to analyze, streamlining the identification process. Additionally, integrating multiomics approaches enables a comprehensive examination of the platelet proteome, lipidome, and metabolome, providing a holistic understanding of platelet biology. This multifaceted analysis is critical for elucidating the complex mechanisms underpinning platelet function and dysfunction. Ultimately, these insights are crucial for advancing therapeutic strategies and improving diagnostic tools for platelet-related disorders and cardiovascular diseases. The integration of multi-omics technologies is paving the way for a deeper understanding of platelet mechanisms, with significant implications for biomedical research and clinical applications.

## 1 INTRODUCTION

Platelets, with diameters ranging from 2 to 4 μm, play a pivotal role in maintaining hemostasis and blood coagulation. Inherited platelet dysfunctions can lead to bleeding disorders or an elevated risk of thromboembolic events such as strokes and cardiovascular conditions [1]. Consequently, platelets are one of the leading causes of mortality worldwide. A comprehensive understanding of platelet functions in hemostasis and their involvement in pathophysiological events such as inflammation, cancer dissemination, and thrombosis necessitates the application of advanced proteomic methodologies across relevant research disciplines [2]. Additional insights into the molecular biology of platelets are crucial to support drug development and identify diagnostic indicators of platelet activation. Posttranslational processes, notably proteolysis and phosphorylation, play a pivotal role in platelet activation [3]. The precise probing and identification of these posttranslational pathways are facilitated by the use of multi-omics technology and biochemical methods. During isolation, platelets exhibit high sensitivity to changes in the natural circulation environment in response to factors such as temperature, pH, and shear stress. Consequently, it is imperative to validate omics data after conducting a thorough investigation into platelet functionality [4]. The absence of a nucleus in platelets significantly reduces the number of proteins present, streamlining proteomics and metabolomics methods based on mass spectrometry. Investigations into platelet phenotypic characteristics and the differential expression of genes have utilized genomic sequencing, genome-wide association studies, and transcriptomics. This review provides a comprehensive overview of both established and innovative multi-omics approaches applicable to the investigation of platelet biology under both healthy and pathological conditions.

## 2 CHEMOPROTEOMIC METHODS FOR COVALENT STRUCTURE DISCOVERY

Covalent chemical indicators, small molecules adept at tagging proteins based on specific characteristics such as enzymatic activity or ligand binding, offer a precise means to investigate the intricate posttranslational signaling processes activated by platelets [5-20]. Employing mass spectrometry alongside specially designed chemical probes allows for a detailed examination of proteolytic actions, particularly those involving the cleavage of peptide bonds by proteases. The activation of platelet proteases, a crucial component in these processes, is orchestrated through various mechanisms, including zymogen transformation, (auto)proteolysis, conformational changes, autoinhibition removal, and increases in the cytosolic Ca^2+^ concentration. The internationally recognized proteolytic signaling pathway in platelets involves the G protein-coupled receptors PAR-1 and PAR-4, which are activated through cleavage by proteases such as thrombin and matrix metalloproteinases (MMPs) [21]. This cleavage triggers subsequent signaling events, including the activation of protein kinases and an increase in the cytosolic Ca^2+^ concentration [22]. An increase in the Ca^2+^ concentration affects intracellular proteins, including those of the cytoskeleton, such as the cysteine protease calpain [23, 24]. The release of MMP isoforms from platelets, which cleave proteinase-activated receptor 1 (PAR-1) and PAR-4, can enhance activation [10]. Despite the limited protein production by platelets, the protein-degrading proteasome complex appears to be involved in platelet signaling and function [11]. Covalent chemical sensors that interact with the active site of a nucleophile through a mechanism-based reaction have elucidated the functions of specific proteases, paving the way for a dynamic understanding of protease control [14]. The following general mechanisms of action of platelets are described below.

Platelets, also known as thrombocytes, are critical components of the hemostatic process and prevent excessive bleeding and facilitate vascular repair. Their life cycle and functionality unfold in distinct phases, starting from their origin in the bone marrow to their active participation in clot formation. During the process of platelet maturation, platelets originate from megakaryocytes, large cells in the bone marrow. The development of megakaryocytes is primarily stimulated by thrombopoietin (TPO) in combination with other growth factors. These cells undergo endomitosis, a unique process in which DNA replicates multiple times without actual cell division, resulting in large, polyploid cells. As they mature, megakaryocytes extend long cytoplasmic protrusions called proplatelets into the blood vessels of the bone marrow. The shear forces of circulating blood help to fragment these proplatelets, releasing individual platelets into the bloodstream. Once released, platelets circulate as anucleate cells primed for activation in response to vascular injury.

The activation of platelets can occur through two main mechanisms: physical injury and chemical signaling. Physical injury, such as the rupture of a blood vessel, exposes the underlying collagen and other matrix proteins to which platelets adhere, initiating their activation. Chemical stimuli also play a crucial role; substances such as thrombin, adenosine diphosphate (ADP), thromboxane A2 (TXA2), and serotonin can bind to specific receptors on the surface of platelets, triggering their activation. The Activation Process and Intracellular Signaling: Upon activation, several rapid changes occur within platelets. They transform from a smooth, discoid shape to a more activated, spherical form with extended filopodia, increasing their contact area with other platelets and the vascular wall. This morphological change is accompanied by the release of dense granules and alpha granules that contain coagulation factors and other molecules essential for the clotting process. These granules enhance the coagulation cascade and help in recruiting more platelets to the injury site. Moreover, activated platelets express fibrinogen receptors (integrin αIIbβ3), allowing them to link together *via* fibrinogen molecules, forming a primary hemostatic plug. The intracellular signaling pathways activated during this process, such as those involving phospholipase C (PLC) and protein kinase C (PKC), lead to an increase in intracellular calcium levels, which are crucial for sustaining platelet activation and stable clot formation.

These processes illustrate the complex and finely tuned mechanisms by which platelets are generated, activated, and contribute to hemostasis, demonstrating their indispensable role in maintaining vascular integrity and responding to vascular damage [14]. The regulation of platelet signaling by posttranslational modifications (PTMs) is shown in Fig. (**1**).

**Fig. (2):**
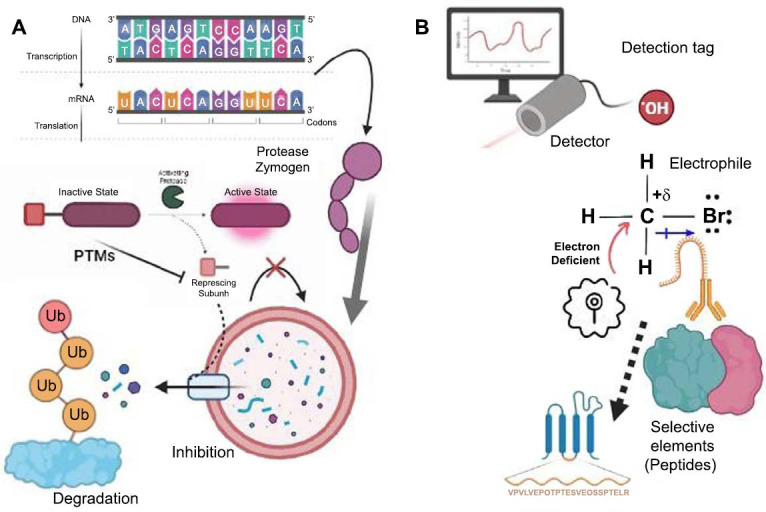
Protease detection [17, 19]: (**A**) Many proteases are initially found in an inactive form called zymogens. They become active through processes such as proteolysis or changes in their shape. Posttranslational modifications (PTMs) and natural inhibitors can influence the active forms of these proteases. Furthermore, active proteases are often regulated or broken down. (**B**) Chemical probes designed to study protease activity typically comprise three components: (1) an electrophilic compound that reacts with the active site's nucleophilic group (found in serine, cysteine, or threonine proteases), (2) a selectivity element, often a peptide that mimics the substrate preferences of the target protease, and (3) a detection tag. This detection tag may take various forms, such as a fluorescent marker for microscopic observation, biotin for purification, a radioactive isotope for imaging, or a bioorthogonal handle such as an alkyne or azide. (The figure was created by the Biorender tool).

Advanced mass spectrometry (MS)-based proteomic techniques, coupled with covalent chemical sensors, enable the analysis of enzyme activity, extending to kinases and phosphatases. This methodology has provided profound insights into disease-related mechanisms and revealed potential therapeutic targets over the last decade [15, 16]. While chemical proteomics applications for platelet biology are relatively scarce, noteworthy studies have utilized chemical probes to study nucleotide-binding proteins, discover inhibitors for serine hydrolases, and create selective inhibitors for platelet-activating factor acetyl hydrolases [17, 19]. These examples underscore the immense potential of chemical proteomics in unraveling the complexities of platelet biology (Fig. **2**).

## 3 IMPLEMENTATIONS OF OMICS TECHNOLOGIES

In the pursuit of unraveling the intricate dynamics of platelet behavior under both physiological and pathological conditions, a multifaceted approach is paramount. Various omics technologies serve as indispensable tools in this exploration, each contributing unique dimensions to the comprehensive understanding of platelet mechanisms. Lipidomics delves into the intricate lipid composition of platelets, shedding light on their structural intricacies. N-terminomics offers insights into protein maturation and turnover by focusing on the identification and quantification of protein N-termini. Glycoproteomics investigates the glycosylation patterns of platelet proteins, revealing crucial details about their roles in signaling and adhesion processes. Global proteomics provides a holistic examination of the entire protein complement, offering a panoramic view of the platelet proteome and its alterations. Phosphoproteomics delves into phosphorylation events, revealing the signaling cascades that regulate platelet activation. Finally, transcriptomics analyses the complete set of RNA transcripts within platelets, providing insights into the genetic underpinnings of their behavior. The integration of these diverse omics methodologies presents a powerful approach to comprehensively decipher the complex and nuanced realm of platelet mechanisms, contributing to advancements in both basic science and clinical research.

### 3.1 Transcriptomics

Despite having no DNA because they are anucleate cell fragments, platelets do have a complex transcriptome that includes mRNA, miRNA, long noncoding RNA, pre-mRNA, and circular RNA species [2]. During proplatelet shedding, a process known as thrombopoiesis, megakaryocyte precursor cells produce several genetic transcripts. Furthermore, there is experimental evidence that mRNA forms can be obtained through yet-to-be-discovered cell transfer mechanisms. Additionally, platelets are capable of converting pre-mRNAs into mRNAs, which may then be translated into proteins. The platelet transcriptome is dynamic even when DNA is not present. It changes by utilizing different initial and final sites, exon skipping, and intron retention in response to external stimuli such as inflammatory signals, pathogen encroachment, and tumor metastasis [25]. The Blueprint collaboration developed one of the largest databases by genome-wide cataloging of RNAs in different kinds of blood cells to gain greater insights into the platelet transcriptome [26]. The current study on platelets and megakaryocytes revealed 57,849 transcripts, suggesting new avenues for understanding the makeup and biological role of platelets [27]. By this investigation, Hung and colleagues examined the pattern of expression for all expressed gene-linked transcripts and discovered that approximately 20,000 of them were found in platelets and/or megakaryocytes at meaningful levels. A total of 14,800 protein-coding transcripts and 5,200 platelet-expressed proteins were categorized, and the results showed a rather uniform distribution pattern across UniProt-based protein localization and function classes [28].

### 3.2 Protein Profiling and Phosphoproteomics

All the proteins that a biological system produces at a specific moment and under specific circumstances are referred to as the proteome [29]. Due to the large diversity of protein abundances, elucidating the whole proteome of a biological system, such as platelets, is difficult. Additionally, posttranslational modifications (PMTs) increase the complexity of the proteome and increase the difficulty of data analysis. Recently, advances in mass spectrometry-based protein characterization have enabled faster and more sensitive detection of more proteins, partially overcoming complex issues [3]. According to the most recent and thorough platelet proteome analysis, nearly 4000 proteins were found [4]. Fortunately, we anticipate that it will be possible to achieve greater coverage of the platelet proteome shortly after the introduction of more advanced proteomics acquisition techniques, such as data-independent acquisition (DIA) in combination with parallel accumulation-serial fragmentation (PASEF). PASEF refers to a technique where data are gathered without targeting specific molecules, alongside a method where ions are accumulated in parallel and then fragmented sequentially. This approach enhances the efficiency and speed of mass spectrometric analysis. This will offer us a better understanding of previously unknown critical protein players in platelet activation processes and platelet-derived biomarkers for illnesses connected to platelets alongside additional disorders. Protein phosphorylation is a rapid PTM-mediated modification in activated platelets. A range of biochemical and signaling pathways can be controlled by protein phosphorylation since it is a reversible process that involves complicated interactions between protein kinases and phosphatases.

**Fig. (3):**
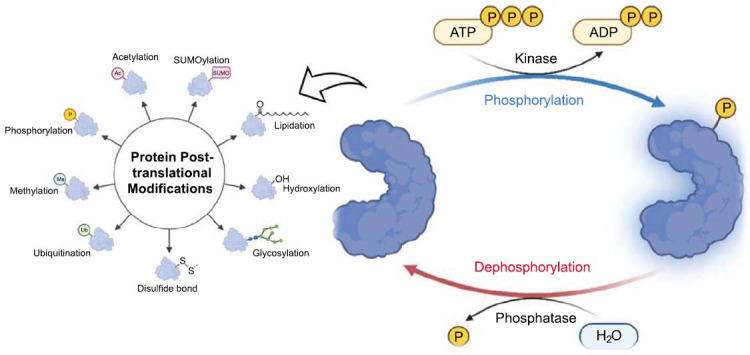
Overview of PTMs in the Phosphorylation Process [30]: Phosphorylation, a prevalent posttranslational modification (PTM), entails the addition of phosphate groups to proteins, imparting regulatory control by inducing changes in their conformation or interactions. This ubiquitous modification significantly impacts diverse cellular processes, including signal transduction and gene expression, underscoring its pivotal role in orchestrating intricate cellular responses. (The figure was created by the Biorender tool).

The comprehensive ensemble of proteins generated by a biological system at a specific moment and under particular circumstances is collectively termed the proteome [29]. The complexity of protein abundances within a biological system, such as platelets, coupled with the intricate landscape of posttranslational modifications (PTMs), poses a challenging task for elucidating the entirety of the proteome. However, recent advancements in mass spectrometry-proteomic methods have significantly accelerated and enhanced the detection of a broader spectrum of proteins, partially alleviating the challenges associated with complexity [30]. Elucidating the entirety of the proteome in platelets is a challenging task due to the large diversity of protein abundances and the complexity introduced by posttranslational modifications (PTMs). Fortunately, recent advancements in mass spectrometry have accelerated the detection of a broader spectrum of proteins. For instance, a comprehensive platelet proteome analysis has identified nearly 4000 proteins. These findings suggest that continued advancements in acquisition techniques, such as PASEF, will further increase our understanding of the platelet proteome and reveal previously unknown critical proteins involved in platelet activation and disease pathways [31]. This anticipated progress will deepen our insights into previously undiscovered pivotal protein players in platelet activation processes and reveal potential platelet-derived biomarkers associated with various platelet-related and additional disorders. Protein phosphorylation is a rapid posttranslational modification observed in activated platelets. Given its reversible nature and intricate interactions between protein kinases and phosphatases, protein phosphorylation exerts control over a multitude of biochemical and signaling pathways (Fig. **3**).

**Fig. (4):**
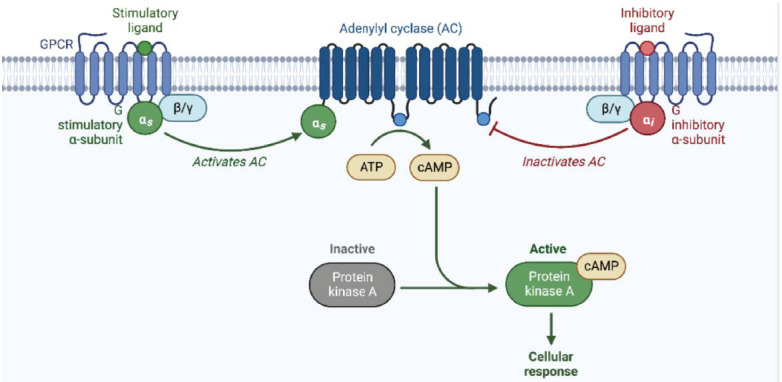
Activation of the PKA (protein kinase-A) pathway [37, 38]: Elevated cAMP levels, resulting from a surge in intracellular signaling, stimulate PKA, a critical regulator of cellular responses. Upon activation, PKA initiates a dynamic cascade of downstream signaling events orchestrating intricate cellular processes. This pathway plays a pivotal role in mediating responses to extracellular signals and modulating various cellular functions, ultimately contributing to the regulation of physiological and biochemical activities within the cell. (The figure was created by the Biorender tool).

Serine, threonine, and tyrosine are typically phosphorylated in substoichiometric amounts in proteins, necessitating the reliance of phosphoproteomic analyses primarily on phosphopeptide enrichment methods, predominantly at the peptide level [32]. To date, metal oxide affinity chromatography (MOAC) has been the most widely utilized method for enriching platelet phosphopeptides. The frequent use of titanium dioxide beads for enrichment, subsequent hydrophilic interaction liquid chromatography (HILIC), and quantification employing tandem mass tags (TMTs) or isobaric tags for relative and absolute quantification (iTRAQ) have established methodologies [33] and quantification utilizing tandem mass tags (TEMs) [34] or isobaric tags for relative and absolute quantification (iTRAQ) [33]. Through the comparative analysis of samples from healthy and ill patients, this approach facilitated the identification of more than 3000 unique phosphopeptides in platelets, revealing previously unidentified activation and inhibition mechanisms [23, 35, 36]. Swieringa and colleagues recently investigated the phosphorylation processes in platelets from individuals with Albright hereditary osteodystrophy syndrome (AHO) [36]. The GNAS complex gene, encoding the GTPase component Gsa, is associated with this syndrome [37, 38]. As a result, platelets from individuals with AHO syndrome are expected to exhibit abnormalities in protein kinase A (PKA)-mediated protein phosphorylation. A total of 2516 phosphorylation sites were discovered during platelet analysis utilizing either iTRAQ- or TMT-based phosphoproteomics, with 453 being under the control of the Gsa-PKA pathway (Fig. **4**). This research revealed novel molecular connections to this pathway. The integration of automated sample preparation and liquid handling devices is expected to significantly enhance the throughput of platelet phosphoproteomic analysis, even though isotopic label techniques have been employed for medium-scale throughput [39]. Anticipated advancements suggest that label-free phosphoproteomics will be particularly beneficial in automated peptide enrichment. With such a platform, the number of samples will cease to be a constraint, enabling the simultaneous processing of up to 96 samples. This is poised to yield improved statistical analysis and potentially more precise targets. A comparable automated procedure has already been applied to hippocampal neurons, revealing a comprehensive phosphoproteome.

### 3.3 N-terminomics and Glycoproteomics

Proteolytic cleavage of proteins by proteases is another posttranslational modification (PTM) implicated in platelet activation. “Terminomics” encompasses a suite of proteomic techniques designed to unveil the substrates of proteases, with a predominant focus on enriching neo-N-termini generated during cleavage events (N-termini). Given the substoichiometric nature of proteolytic processes, enrichment strategies are imperative to ensure the distinct separation of the original and neo-N-termini of cellular proteins from those produced by tryptic digests.

Several existing proteomic enrichment methods, such as charge-based fractional diagonal chromatography (ChaFRADIC), terminal amine isotopic labeling (TAILS), combined fractional diagonal chromatography (COFRADIC) [1], and subtilase-N-terminal biotinylation, have been employed. Positive selection methods, such as selective biotinylation before tryptic digestion, or negative selection techniques, such as initial blocking of desired primary amines (*e.g*., through demethylation) followed by the removal of undesirable tryptic peptides, are utilized for sample enrichment. These techniques can be applied to analyze up to eight samples, leveraging technologies such as iTRAQ or dimethylation for multiplexing and direct comparison of three samples. Multiplexing quantification techniques have been implemented for both TAILS and ChaFRADIC [1]. Prudova and colleagues [1] delved into how proteolytic processing influences the preservation of platelet concentrates, which, despite their therapeutic utility, have a short shelf life [1]. To elucidate the intricate molecular mechanisms and biomolecular changes associated with platelet activation or inhibition, multi-omics techniques integrate various omics datasets. To elucidate poorly understood molecular pathways underlying platelet procoagulant activity, Solari and colleagues reported the first multiomics iTRAQ-based combined proteomic method [23]. Using the TAILS method, the authors assessed platelet protein maturation, posttranslational Nα-acetylation, N-terminal methionine excision, and proteolytic processing. Intriguingly, metalloproteinases were found to predominantly regulate proteolytic processing during storage, providing fresh insights into how extra cytosolic proteases impact protein integrity and platelet function during storage. Another crucial PTM related to platelet functions in hemostasis and vascular integrity is the glycosylation of membrane proteins. Glycoproteomic investigations will further elucidate platelet interactions with other blood and vascular cells [22]. Platelet glycoproteomic investigations are anticipated to significantly benefit the elucidation of platelet interactions with other blood and vascular cells. Existing glycoproteomic methods have primarily relied on either hydrazide affinity capture for N-glycosylation [30] or concanavalin A affinity purification for lectin-based glycopeptide enrichment [31] to capture glycopeptides from isolated platelets. In a notable example of glycoproteomics, Shah and colleagues explored the platelet glycoproteome in response to aspirin stimulation, revealing important insights into platelet glycoprotein dynamics. The study used methods like hydrazide affinity capture for N-glycosylation, offering valuable data on platelet glycosylation patterns [30], or the following methods, a requisite step involving separating glycan motifs from peptides using PNGaseF before proteolytic processing, enabling the identification of glycosylations through LC-MS/MS analysis [31] to capture glycopeptides from isolated platelets.

### 3.4 Metabolomics

There are many metabolites and metabolic pathways that influence platelet reactivity in different ways. While metabolomics offers a detailed analysis of particular classes of biomolecules, there is no single approach that covers the entire metabolome [40].

Historically, metabolomics has evolved from classical biochemistry by allowing the routine analysis of hundreds to thousands of small molecules. Advances in nuclear magnetic resonance (NMR) and mass spectrometry (MS) techniques have improved detection limits down to the femtomole range in metabolomics analysis. Upon acquisition of the data (post hoc), data analysis using bioinformatics approaches is required to identify metabolites [41].

Following metabolite assignment, experienced statistical handling and interpretation of big omics data are necessary to fully explore the capacity of metabolomics [42]. The combination of metabolomics and systems biology offers predictive tools for understanding cardiovascular disease (CVD). For example, metabolomics can serve as a surrogate marker for platelet activation during cardiovascular events, which opens up possibilities for prediagnostic applications. One recent report highlighted that untargeted metabolomics yielded approximately 2,000 metabolites, of which more than 400 were identified to play a role in a human metabolic pathway [42]. The authors were able to associate approximately 22% to be significantly associated with at least one readout of platelet reactivity. Most associations involved lipids, especially prostaglandins and leukotrienes, which are members of the eicosanoid family. Interestingly, polyphenols derived from dietary sources have also been shown to inhibit the reactivity of platelets.

When metabolomics meets systems biology requirements, it becomes a predictive tool. Metabolomic analysis for dissemination, e.g., the use of surrogate markers of platelet activation, can contribute significantly to our understanding of CVD, which can be attributed to platelet activation, by providing future potential prediagnostic tools [43].

### 3.5 Lipidomics with Clinical Implications

Lipidomics encompasses mass spectrometry-based methods aimed at discovering and quantifying molecular species of lipids, whether free or interacting with other molecules. In addition to influencing platelet activation or inhibition, variations in phospholipid composition may serve as distinguishing factors between healthy and ill individuals [20]. Peng and colleagues identified 400 lipid species, including phospholipids and eicosanoids, in their analysis of the platelet lipidome. The authors compared the lipidomics network of resting and activated murine platelets, a finding later confirmed in human platelets [20, 21]. Surprisingly, less than 20% of the platelet lipidome undergoes alterations during activation, primarily affecting phospholipid molecular species containing arachidonic acid [22]. This discovery paves the way for further exploration of lipid-related platelet functions [20]. A recent lipidome investigation of murine platelets with a genetically engineered altered lipid profile characterized by modestly elevated plasma lipid and cholesterol levels linked to high-fat meal intake revealed significant differences in lipid species, notably elevated levels of free cholesterol and cholesteryl esters [23]. By comparing control platelets with platelets from a patient with Scott syndrome, a rare hereditary bleeding disorder marked by ANO6 mutations and dysfunction of the platelet procoagulant response mechanism, the authors observed alterations in the global proteome, phosphoproteome, and N-terminus. Using a combined proteome and phosphoproteome approach, Solari and colleagues quantified 2278 distinct proteins and 1566 phosphopeptides in stimulated control and patient platelets [21, 23]. This study provided unique insights into the Ca2 dependence of platelets and the anoctamin-6 and calpain-related platelet activation pathways when stimulated by thrombin, convulxin, or the Ca2 ionophore ionomycin [24]. Western blot analysis confirmed the absence of anoctamin-6 in the patient's platelets [24]. Additionally, by measuring N-terminal peptides, the study elucidated the role of calpain in the platelet procoagulant response. In conclusion, this multi-omics investigation has laid the foundation for further research on patient platelets in various diseases.

Recent studies exploring platelet lipidomics and metabolomics have provided key insights into cardiovascular outcomes. In patients with symptomatic coronary artery disease, the level of platelet oxidized low-density lipoprotein (LDL) detected by flow cytometry was elevated, especially in those with acute coronary syndrome and angiographic evidence of intracoronary thrombi. Oxidized low-density lipoprotein (ox-LDL) is a form of oxidized LDL cholesterol. oxLDL is known to play a significant role in the development of atherosclerosis, which can lead to coronary artery disease. ox-LDL is thought to be more atherogenic than normal LDL because it can induce inflammation in the arteries, leading to plaque buildup. Platelets and oxLDL, Typically, platelets are small blood cells involved in clot formation to prevent bleeding. Research has suggested that platelets can interact with oxLDL, and this interaction may play a role in the development and complications of atherosclerotic disease, including acute coronary syndrome (ACS). Flow cytometry is a technique often used to analyze the physical and chemical characteristics of particles in a fluid as it passes through at least one laser. It can detect and measure the levels of ox-LDL associated with or bound to platelets. This measurement can be particularly important in identifying the extent of platelet activation and its potential contribution to thrombus formation (clot formation inside a blood vessel) [43]. Importantly, lipidomic analysis revealed increased intraplate-let levels of oxidized phospholipids, cholesteryl esters, sphingomyelin, ceramides, di-triacylglycerols, and acylcarnitines in patients with coronary artery disease compared with age-matched controls, suggesting that an altered platelet lipidome might predispose patients with coronary artery disease to thrombosis [44]. These results were confirmed in a large cohort of patients (n=1057) with coronary artery disease, which showed that upregulated platelet phospholipids and fatty acids were associated with altered platelet function *in vitro* and independently predicted adverse cardiovascular events [44]. These observations may be related to the release of biologically active phospholipids into the bloodstream, leading to thrombosis and/or angiogenesis [45, 46]. Ultimately, using platelet lipidomics and metabolomics. These findings underscore the potential of platelet lipidomics and metabolomics for improved risk stratification in cardiovascular diseases.

## CONCLUSION

Over the past year, significant strides have been made in understanding platelet biology through diverse omics technologies. Covalent chemical indicators, mass spectrometry, and chemical probes have revealed that proteolytic actions are crucial for platelet activation. The integration of lipidomics, N-terminomics, glycoproteomics, global proteomics, phosphoproteomics, and transcriptomics has yielded a comprehensive view of platelet mechanisms. Despite lacking a nucleus, platelets exhibit a dynamic transcriptome in response to external stimuli. Mass spectrometry-based proteomics has identified thousands of proteins, with ongoing advancements promising greater coverage. Protein phosphorylation, terminomics, and metabolomics analyses have unveiled intricate details of platelet functions and their role in diseases, contributing to predictive models for cardiovascular diseases. Recent studies linking altered platelet omics to cardiovascular outcomes highlight the potential for improved risk stratification. In essence, this progress underscores the transformative impact of omics technologies on platelet research, offering insights into therapeutic targets and diagnostic tools for platelet-related disorders and cardiovascular diseases.

## Figures and Tables

**Fig. (1):**
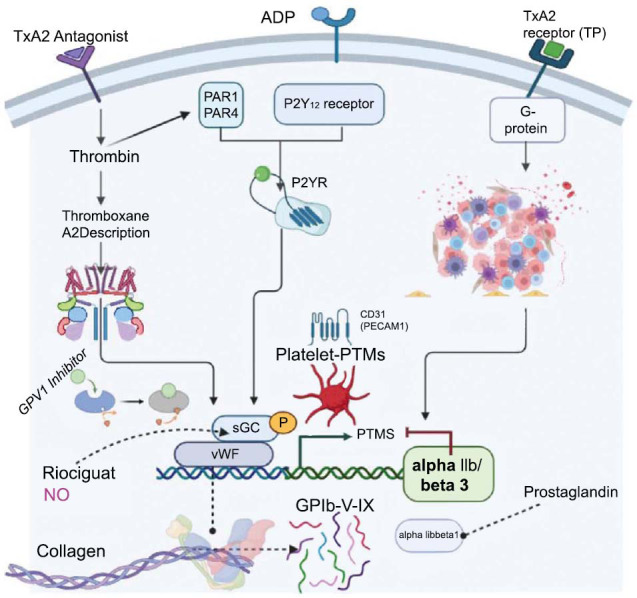
Platelet Signaling Regulated by Posttranslational Modifications (PTMs) [14]: Platelet activation and inhibition are controlled by alterations in PTMs, specifically glycosylation and phosphorylation. When platelets adhere to a damaged vessel wall, they transition from a discoidal to a semi round shape with filopodia. Upon exposure to agonists such as collagen and thrombin, activated platelets with filopodia release autocoids such as ADP and thromboxane. Within the cell cytosol, numerous protein kinases and phosphatases become active, initiating phosphorylation and dephosphorylation events on their target proteins. Additionally, depending on the state of platelet activation, proteases are activated and cleave their protein substrates. Moreover, modifications to platelet lipids and glycan composition play a role in the mechanisms of platelet activation (The figure was created by the Biorender tool).

## LIST OF ABBREVIATIONS

ADP: Adenosine Diphosphate
MMPs: Matrix Metalloproteinases
PKC: Protein Kinase C
PLC: Phospholipase C
TPO: Thrombopoietin
TXA2: Thromboxane A2
